# Endovascular pulmonary arterial fiducial marker placement for particle radiotherapy using proton or carbon-ion beams for lung cancer

**DOI:** 10.1093/jrr/rrag022

**Published:** 2026-04-23

**Authors:** Kazuki Terashima, Tomoyuki Gentsu, Daiki Takahashi, Yoshiro Matsuo, Yasue Niwa, Yusuke Demizu, Sunao Tokumaru, Tomoaki Okimoto

**Affiliations:** Department of Radiology, Hyogo Ion Beam Medical Center, 1-2-1 Kouto, Shingu-cho, Tatsuno, Hyogo 679-5165, Japan; Department of Radiology, Kobe University Graduate School of Medicine, 7-5-2, Kusunoki-cho, Chuouku, Kobe, Hyogo 650-0017, Japan; Department of Radiology, Hyogo Ion Beam Medical Center, 1-2-1 Kouto, Shingu-cho, Tatsuno, Hyogo 679-5165, Japan; Department of Radiology, Hyogo Ion Beam Medical Center, 1-2-1 Kouto, Shingu-cho, Tatsuno, Hyogo 679-5165, Japan; Department of Radiology, Hyogo Ion Beam Medical Center, 1-2-1 Kouto, Shingu-cho, Tatsuno, Hyogo 679-5165, Japan; Department of Radiation Oncology, Hyogo Ion Beam Medical Center Kobe Proton Center, 1-6-8, Minamicho, Minatozima, Chuouku, Kobe, Hyogo 650-0047, Japan; Department of Radiology, Hyogo Ion Beam Medical Center, 1-2-1 Kouto, Shingu-cho, Tatsuno, Hyogo 679-5165, Japan; Department of Radiology, Hyogo Ion Beam Medical Center, 1-2-1 Kouto, Shingu-cho, Tatsuno, Hyogo 679-5165, Japan

**Keywords:** fiducial marker, endovascular, particle therapy, lung cancer

## Abstract

This study evaluated the safety, feasibility and efficacy of endovascular pulmonary arterial fiducial marker (EVFM) placement for image-guided particle therapy using proton or carbon-ion beams in patients with primary or metastatic lung cancer. Methods: A 5F catheter system was used to insert platinum coil markers via the right femoral vein into a distal pulmonary artery near the tumor under fluoroscopic guidance. Tumor-to-marker distances, marker migration and therapy completion were assessed. From July 2016 to January 2019, 54 patients (38 men, 16 women; median age, 71 years) with 46 primary and 8 metastatic lung tumors underwent marker placement. Tumors were located in 33 right and 21 left lobes, with a median diameter of 22 mm. Ten patients received proton therapy, and 44 received carbon-ion therapy. There were no marker placement related complications. Transient arrhythmias occurred only when the guide wire passed through the right ventricle. Tumor-to-marker distances ranged from 0–17 mm (median, 5 mm). Migration during irradiation was minimal (median, 0 mm); one case showed a 4-mm shift due to lung deformation, not marker displacement. All patients completed scheduled therapy without marker migration. EVFM placement is a feasible and safe alternative to computed tomography- or bronchoscopy-guided fiducial marker placement, offering accurate localization with minimal complications or marker migration in particle therapy for lung cancer.

## INTRODUCTION

Radiation therapy for lung cancer (primary lung cancer, lung metastasis) has progressed from conventional radiotherapy to three-dimensional conformal radiotherapy and eventually to stereotactic body radiotherapy (SBRT) and intensity-modulated radiotherapy [1, 2]. These advanced radiation therapies are radical because they irradiate the tumor with a high dose of radiation, so it is necessary to accurately identify the location of the tumor by using an image-guided radiotherapeutic technique [3]. Similarly, particle therapy using proton or carbon-ion beams, which have been recently developed as innovative radiotherapeutic approaches, also requires high-precision irradiation to achieve strict dose distribution [4, 5].

For these high-precision radiotherapies, the patient’s body must be accurately fixed to the treatment bed, and respiratory suppression or respiratory synchronization must be performed. More importantly, the beam should be aligned with the tumor by accurately detecting the location of the tumor. For this purpose, fiducial markers are commonly placed in the vicinity of the tumor [3, 6].

Generally, two methods for placing fiducial markers, CT-guided percutaneous and bronchoscopy-guided placement, have been widely performed, but each has several problems. Although CT-guided placement can easily insert fiducial markers at the target location, pneumothorax has been reported as a complication in 56% of cases and chest drainage was required in 2% to 22% [7–10]. Moreover, marker migration was seen in 5% to 9% of cases. In transtracheoscopic placement, the incidence of pneumothorax has ranged from 0% to 6%, which is less than that for CT-guided placement, but the marker migration rates of 1% to 50% and complications due to general anesthesia are serious problems [11–14].

As an alternative to these placement methods, Prevost *et al*. [15] reported the effectiveness of using an endovascular catheter to place markers in the pulmonary artery. No serious complications and less migration were reported, so the placement technique was considered to be promising. This study aim was to assess the merits and disadvantages of endovascular pulmonary arterial fiducial marker (EVFM) placement.

## PATIENTS AND METHODS

### Eligibility

Patients who received EVFM and particle therapy using a proton beam or a carbon-ion beam for primary lung cancer or metastatic lung cancer were retrospectively analysed. Patients who obtained informed consent and had no severe heart failure or arrhythmia evaluated by a cardiologist were eligible. Details of the eligibility criteria for particle therapy for primary and metastatic lung cancer have been described in previous reports [16]. This study was conducted with the approval of the Ethics Committee of the institution.

### EVFM placement

**Fig. 1:**
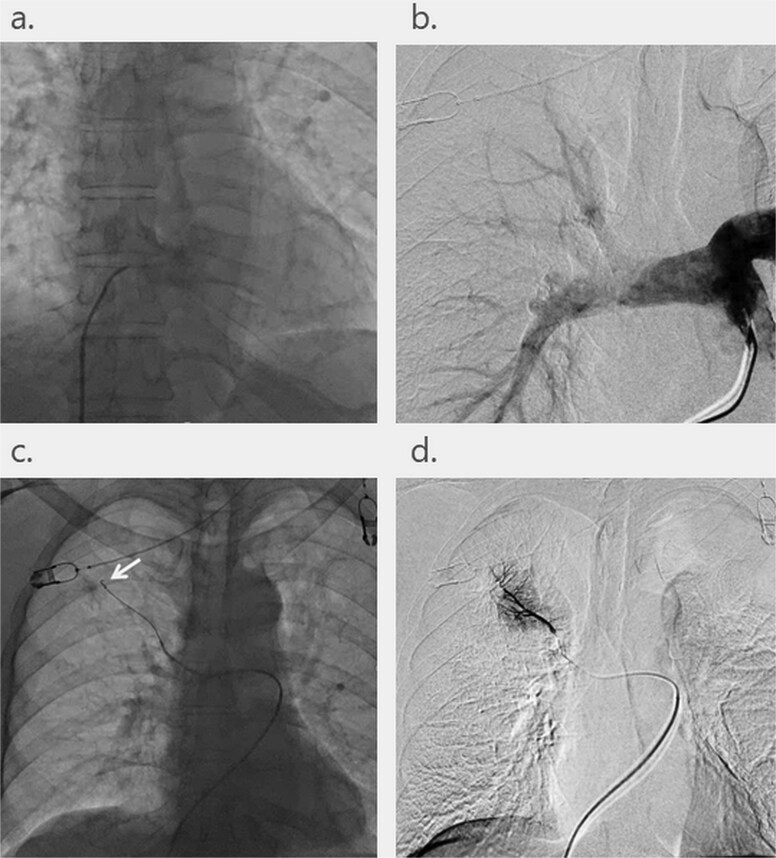
Technique of transcatheter pulmonary arterial fiducial marker placement. (a) A guiding catheter is inserted into the inferior vena cava via the right femoral vein with ultrasound echocardiography guidance. (b) A 6Fr headhunter-type catheter is placed into the pulmonary artery via the right atrium and ventricle through a guiding catheter. (c) An embolic platinum coil is placed as a fiducial marker into a peripheral pulmonary artery near the tumor by using a micro-catheter through a guiding catheter. (d) Digital subtraction angiography from the micro-catheter.

The right femoral vein was punctured under echo guidance with local anesthesia and no sedation. A 5F catheter (HAA; MEDIKIT, Tokyo, Japan) with 0.035-inch guide wire (SSS; Technowood, Tokyo, Japan) was inserted into the pulmonary artery via the inferior vena cava, right atrium and right ventricle through a dedicated 4.5F guiding sheath (Parent Plus; Medikit, Tokyo, Japan) three-dimensionally pre-shaped for EVFM. Angiography was always performed to confirm that the catheter tip was inserted into the pulmonary artery. Thereafter, a vascular embolization platinum coil (FPC18 complex helical 2 mm × 10 mm; Boston Scientific Japan, Tokyo, Japan) was pushed out as an EVFM into a vessel through a micro-catheter (Sumius LEONIS Mova, Sumitomo Bakelite, Tokyo, Japan) advanced to the periphery of the pulmonary artery near the tumor by using a 0.016-inch micro-guide wire (Meister, Asahi Intecc, Aichi, Japan). After removing the guiding sheath, the punctured wound was compressed for 30 seconds, and then, the patient rested on the bed for 2 hours (Fig. 1).

### Particle therapy with proton or carbon-ion beam

Treatment planning started within 1 week after the EVFM placement. Each patient was immobilized by using a custom-made thermoplastic cast. Then, 2-mm-thick CT images were obtained during the exhalation phase by using a respiratory gating system. The clinical target volume (CTV) was defined as the gross tumor volume with a 5-mm margin in all directions identified on plain or contrast-enhanced CT images. The planning target volume was defined as the CTV with a setup margin of 5 mm and a 1- to 5-mm respiration motion margin calculated from the measured values of EVFM movement obtained by frontal and lateral dynamic fluoroscopic images, with respiration stabilized by the respiratory gating system. If the EVFM was included in the beam path, dose calculation was performed after replacing the EVFM with soft-tissue density. However, beam directions that avoided passing through the EVFM were selected whenever possible. For each irradiation, the alignment was performed by using the EVFM after collation with the spine. One to four portals were used in the proton or carbon-ion beam. As a respiratory gating system, an AZ-733 (Anzai Medical, Tokyo, Japan) was used for beam irradiation during the exhalation phase. Proton beams of 150 MeV or carbon-ion beams of 320 MeV generated by using a synchrotron accelerator (Mitsubishi Electric Corporation, Tokyo, Japan) were used for treatment.

The proton therapy protocols involved the delivery of total doses of 66-Gy equivalents (GyE) in 10 fractions or 74 GyE in 37 fractions. The carbon-ion therapy protocols specified delivery of total doses of 66 GyE in 10 fractions or 69.6 GyE in 12 fractions. No patients received chemotherapy concurrently with particle therapy.

### End points

**Fig. 2:**
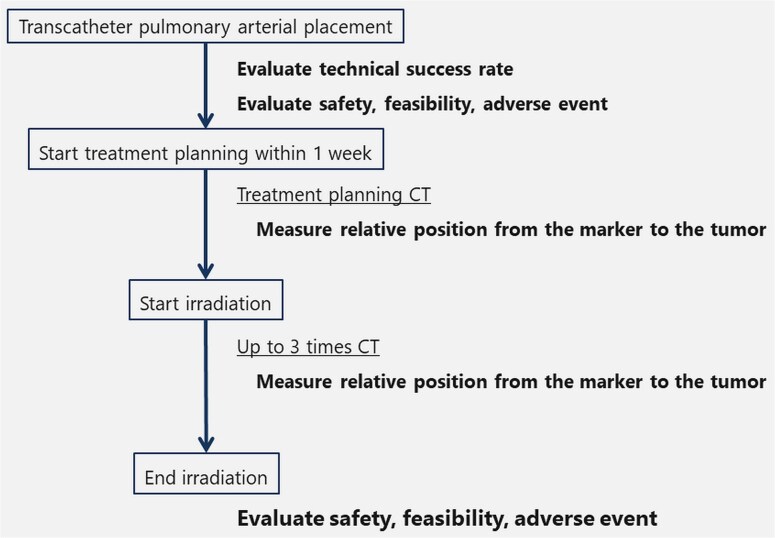
Flowchart of this study.

**Fig. 3:**
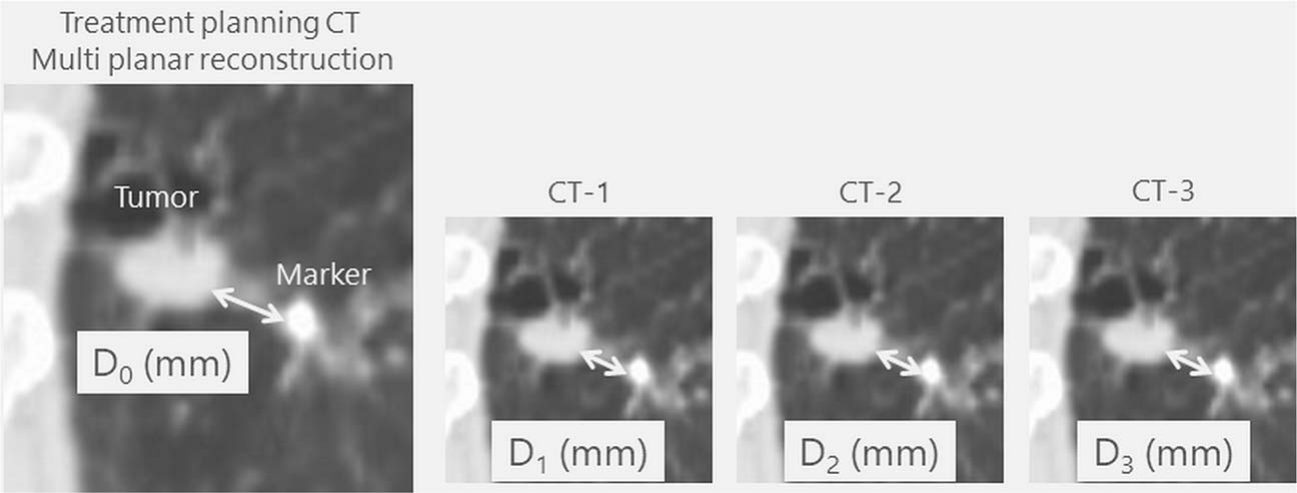
Evaluation methods for measuring relative positions from the marker to the tumor. D_0_, distance from the tumor margin to the marker at treatment planning CT; D1–D3, distances from the tumor margin to the marker in the first to third CT scans, respectively.

Figure 2 shows the evaluation flow of this study. The safety and feasibility of EVFM placement were assessed at the time of the placement, treatment plan start and irradiation end. The technical success rate was defined as the completion of planned particle therapy using the EVFM. The usefulness of the EVFM for particle therapy was examined by measuring the relative distance between the tumor and EVFM using multi-planar reconstruction images of CT at treatment planning (D_0_, mm). The incidence rate of EVFM migration during the irradiation period was evaluated by measuring the relative distance between the tumor and EVFM by performing CT one to three times (D_1–3_, mm) performed weekly during the irradiation period. The migration distance (MD) was evaluated by the average of D_1_–D_0_, D_2_–D_0_ and D_3_–D_0_ (Fig. 3).

Adverse events were evaluated using CTCAE version 4.0. The period from the EVFM placement procedure to the completion of particle therapy was defined as the acute phase, and the period thereafter was defined as the late phase, which was evaluated at each follow-up visit for treatment response assessment after particle therapy.

## RESULTS

### Patient and tumor characteristics

Between July 2016 and January 2019, EVFM placements were performed for a total of 54 patients. The patient characteristics are summarized in Table 1. Thirty-eight patients were male and sixteen were female, with a median age of seventy-one years. Forty-six patients had primary lung cancer, and eight had metastatic lung cancer; thirty-three involved the right lobe; twenty-one, the left lobe; twenty-nine, the upper lobe; one, the middle lobe; and twenty-four, the lower lobe, with a median tumor diameter of 22 mm. Ten patients were treated with proton beam and forty-four patients with carbon-ion beam.

**Table 1:** Patient characteristics

*n* = 54	
Age, range (median)	42–90 (71)
Sex, (*n*)	
Male	38
Female	16
Origin of tumor, (*n*)	
Primary lung cancer	46
Metastatic lung cancer	8
Tumor diameter, range (median) (mm)	5–55 (22)
Lobe, (*n*)	
Right	33
Upper	18
Middle	1
Lower	14
Left	21
Upper	11
Lower	10
Particle beams, (*n*)	
Proton	10
Carbon ion	44
Treatment protocols	
Proton	66–74 GyE in 10–37 fractions
Carbon ion	60–69.6 GyE in 10–12 fractions

Abbreviation: GyE, gray equivalents

### Safety and feasibility

The results are summarized in Table 2. Fifty-four EVFMs were placed in all patients. There were no complications other than transient arrhythmias when the guide wire was passed through the right ventricle. The fluoroscopy time at EVFM placement ranged from 4 to 76 minutes (median, 19.8 minutes), and the amount of contrast agent used ranged from 10 to 90 ml (median, 16 ml). No patients had any complaints such as fatigue or pain during the EVFM placement. In four cases, temporary catheter insertions into the coronary vein via the inferior vena cava or into the pulmonary vein via a patent foramen ovale or atrial septal defect were observed. D_0_ ranged from 0 to 17 mm (median, 5 mm), and all patients were initiated on the particle therapy using these fiducial markers.

**Table 2:** Results

*n* = 54	
Technical success rate, (%)	100
Time of X-ray fluoroscopy, range (median), (minutes)	4–76 (19.8)
Volume of contrast agent usage, range (median), (ml)	10–90 (16)
D_0_, range (median), (mm)	0–17 (5)
Average of D_1_–D_0_, D_2_–D_0_ and D_3_–D_0_, range (median), (mm)	0–4 (0)

Abbreviations: D_0_, relative distance between the tumor and fiducial marker at treatment planning; D_1–3_, relative distance between the tumor and fiducial marker one to three times during the irradiation period.

### Migration

The MD change during the irradiation period ranged from 0 to 4 mm (median, 0 mm). Only one case showed a 4-mm deviation, which was determined to have been caused by lung deformation, not migration by CT analysis; all others were 0 mm.

The technical success rate was 100% because all patients with EVFM were successfully irradiated as scheduled.

## DISCUSSION

In this study, favorable safety, feasibility and a technical success rate of 100%, with minimal toxicity and complication of EVFM placement, were achieved. Therefore, EVFM placement is considered to be a promising modality for fiducial marker placement for particle therapy for lung tumors. To our knowledge, this study is the first report describing the outcomes of EVFM for particle therapy. In addition, because the necessity for fiducial markers is the same for both particle therapy and photon radiation therapy, such as SBRT (including CyberKnife and intensity-modulated radiotherapy), EVFM placement is expected to be useful for photon radiation therapy.

Table 3 shows previous reports of EVFM placement for SBRT using the CyberKnife for lung cancer. Baker *et al*. [17] reported a retrospective comparison of 416 patients with 1335 endovascular-placed fiducial markers and 30 patients with 80 CT-guided percutaneous transthoracic-placed fiducial markers. The EVFM placement resulted in fewer complications, with 0.2% experiencing grade 3 arrhythmia, than those of percutaneous marker placement, with 33% experiencing pneumothorax. Although those authors did not report the incidence of migration of fiducial markers and success rate of radiation therapy, another study by Karaman *et al*. [18] reported that all 14 patients underwent CyberKnife therapy with no major complications and no marker migrations after their EVFM placement. Additionally, Mongeon *et al*. [19] reported a comparison of 15 patients with four to five markers per patient inserted by EVFM placement and 114 patients with three to four markers per patient inserted by CT-guided percutaneous transthoracic placement. They reported that 52.6% of the patients with percutaneous placement had pneumothoraces and 11.4% required chest tube placement, although no pneumothorax occurred at EVFM placement. Moreover, a review report of CT-guided percutaneous transthoracic fiducial marker placement for lung cancers states that pneumothorax occurred in 5% to 67% of all patients, including 3% to 22% chest tube placement, and fiducial marker migration occurred in 5% to 9% [10]. The results of these reports and this study indicate the effectiveness of EVFM placement for lung cancer, with fewer complications and migrations than those of CT-guided percutaneous transthoracic marker placement. In particular, because particle therapy is often performed for patients with poor pulmonary function caused by emphysema or interstitial pneumonia [20], EVFM placement with few complications is considered to be very effective.

**Table 3:** Previous reports of EVFM placement

	Number of patients	Number of coils	Coil type	Radiotherapy	Complications > G3
Karaman 2015	14	49	2–3 mm platinum	CyberKnife	0
Mongeon 2017	15	62	2–3 mm platinum	CyberKnife	0
Baker 2019	416	1335	Tornade	CyberKnife	Arrhythmia 0.2%
This study	54	54	2–10 mm platinum	Particle therapy	0

As another marker placement technique, bronchoscopic fiducial marker placement into the bronchus is generally performed sometimes with assistance of virtual navigational bronchoscopy and endobronchial US. The disadvantages of this technique are considered to be that general anesthesia and sedation sometimes are required, correct placement near the tumor without navigation is difficult, and marker migration often occurs because there is no way to fix the marker to the bronchus wall. Lachkar *et al*. [14] stated that marker migration occurred in 1% to 50% of cases. EVFM placement is considered safer and easier than the bronchoscopic placement, and the lack of a need for general anesthesia, easy hemostasis, and short rest time after removal of the catheter because of vein puncture are advantages of EVFM placement.

We used sigmoidal-shaped vascular embolization coils as markers. Because this type of coil looks like a nonphysiological shape on fluoroscopy, it is highly visible even if it overlaps with the blood vessels, heart, diaphragm, or bone. Further, there are few migrations because of the characteristic sigmoidal shape despite the presence of low-halation artifacts on CT images due to total coil lengths as short as 10 mm. We were able to complete particle therapy in all cases for these reasons, but the automatic tracking system in CyberKnife or other radiotherapeutic methods using motion-tracking may not recognize the fiducial markers because of the low metal mass of this coil. In such cases, as noted in other reports [15, 17–19], larger embolization coils might be needed, but further verification is required for each system.

The problem with EVFM placement is the risk of embolization of the pulmonary artery. In this study, no clinical respiratory dysfunctions were observed, although a small pulmonary infarction may have occurred because of coil embolization at the periphery of the pulmonary artery. In previous reports [15, 17–19], complications due to pulmonary embolism were not explained; therefore, EVFM placement is considered to be safe. However, EVFM placement should be performed with sufficient care because, if the coil is placed at the central pulmonary artery, it may cause a clinical pulmonary infarction. In addition, the placed coil cannot be removed; however, long-term placement of platinum vascular embolization coils is frequently used in other angiography techniques and is not considered to be a problem.

Because irradiation of large doses has been performed in hypo-fractions in recent SBRT and particle therapy using proton and carbon-ion beams for lung cancer, it is very important that every fraction achieves accurate irradiation by detecting inter-fractional motion of the tumor for the delivery of planning doses. Because we only reported the outcomes of treatment with particle therapy using proton or carbon-ion beams for lung cancer before introduction of EVFM placement, it is unclear whether the fiducial marker improved treatment outcome. We are now planning to analyse and report on the effects of particle therapy after EVFM placement.

This study has several limitations. First, this was a single-institution, single-arm retrospective analysis. Randomized controlled trials are required to compare this approach with other techniques. In addition, the visibility of the marker with other radiotherapy systems and its applicability to dynamic tracking have not yet been evaluated. Standardized pre-procedural planning protocols were not established, and transient arrhythmia were not systematically recorded. Moreover, routine imaging to assess for pulmonary infarction was not performed, potentially leading to underdetection of subclinical events. Therefore, multicenter studies using various radiotherapy platforms are necessary to expand its clinical applicability.

## CONCLUSION

The study showed that EVFM placement for particle therapy of lung cancer was feasible and effective and can be considered as an alternative method for fiducial marker placement in patients with lung cancer.
